# Genetic ablation of carbonic anhydrase IX disrupts gastric barrier function via claudin‐18 downregulation and acid backflux

**DOI:** 10.1111/apha.12923

**Published:** 2017-10-19

**Authors:** T. Li, X. Liu, B. Riederer, K. Nikolovska, A. K. Singh, K. A. Mäkelä, A. Seidler, Y. Liu, G. Gros, H. Bartels, K. H. Herzig, U. Seidler

**Affiliations:** ^1^ Department of Gastroenterology Hannover Medical School Hannover Germany; ^2^ Department of Department of Gastroenterology Affiliated Hospital of Zunyi Medical College Zunyi China; ^3^ Institute of Biomedicine and Biocenter of Oulu Oulu University Finland; ^4^ Department of Physiology Hannover Medical School Hannover Germany; ^5^ Department of Anatomy Hannover Medical School Hannover Germany; ^6^ Present address: Department of Thyroid and Breast Surgery Affiliated Hospital of Zunyi Medical College Zunyi 563003 China; ^7^ Present address: Department of Physiological Chemistry University of Halle Hollystr. 1 06114 Halle (Saale) Germany

**Keywords:** carbonic anhydrase, claudins, gastric acid, gastric mucosal defence, pH_i_ regulation, tight junctions

## Abstract

**Aim:**

This study aimed to explore the molecular mechanisms for the parietal cell loss and fundic hyperplasia observed in gastric mucosa of mice lacking the carbonic anhydrase 9 (CAIX).

**Methods:**

We assessed the ability of CAIX‐knockout and WT gastric surface epithelial cells to withstand a luminal acid load by measuring the pH_i_ of exteriorized gastric mucosa *in vivo* using two‐photon confocal laser scanning microscopy. Cytokines and claudin‐18A2 expression was analysed by RT‐PCR.

**Results:**

CAIX‐knockout gastric surface epithelial cells showed significantly faster pH_i_ decline after luminal acid load compared to WT. Increased gastric mucosal IL‐1*β* and iNOS, but decreased claudin‐18A2 expression (which confer acid resistance) was observed shortly after weaning, prior to the loss of parietal and chief cells. At birth, neither inflammatory cytokines nor claudin‐18 expression were altered between CAIX and WT gastric mucosa. The gradual loss of acid secretory capacity was paralleled by an increase in serum gastrin, IL‐11 and foveolar hyperplasia. Mild chronic proton pump inhibition from the time of weaning did not prevent the claudin‐18 decrease nor the increase in inflammatory markers at 1 month of age, except for IL‐1*β*. However, the treatment reduced the parietal cell loss in CAIX‐KO mice in the subsequent months.

**Conclusions:**

We propose that CAIX converts protons that either backflux or are extruded from the cells rapidly to CO_2_ and H_2_O, contributing to tight junction protection and gastric epithelial pH_i_ regulation. Lack of CAIX results in persistent acid backflux via claudin‐18 downregulation, causing loss of parietal cells, hypergastrinaemia and foveolar hyperplasia.

Despite intense research over the last century, the molecular mechanisms of the gastric mucosal barrier function to gastric acid are incompletely understood.^1^ While the pH_i_ regulatory mechanisms of gastric epithelial cells have been characterized in great detail on a cellular and molecular level, their interplay during a luminal acid load *in vivo* and their relevance for gastric mucosal protection are less clear.

In particular, the long‐term effects of normal or even low gastric acidity on a gastric mucosa that harbours an intrinsic defect in the barrier function are largely unknown.^1^, ^2^

Carbonic anhydrases are a family of metalloenzymes that catalyse the hydration of CO_2_ to ${\text{HCO}}_{3}^{-}$ and protons and *vice versa*, thus facilitating the dissipation of pH gradients and the movement of protons or base ions across cell membranes. Carbonic anhydrase IX (CAIX) is a transmembrane carbonic anhydrase isoform that plays an important role in the regulation of the pH microenvironment within tumours, thus facilitating their growth,^3^ and is therefore considered an interesting drug target for antiproliferative therapy.^4^, ^5^ CAIX is weakly expressed post‐natally in most tissues except in tumours. In the gastric mucosa, however, it remains expressed throughout adult life^6^, ^7^ and is localized to the basolateral membrane of the epithelial cells with surface cell predominance.^8^

This localization poses the CAIX directly adjacent to the tight junctions and the basolateral pH_i_ regulators, potentially allowing the latter to extrude acid or import base at a much higher rate by dissipating acidic pH microdomains and enhancing ${\text{HCO}}_{3}^{-}$ availability at the basolateral membrane.^9^

A CAIX‐knockout mouse strain showed noticeable abnormality only in the stomach, with alteration in the glandular morphology.^8^ The authors speculated that CAIX may contribute to the balance between differentiation and proliferation in gastric mucosa via negative control of cell proliferation. However, this hypothesis does not take into account the cellular expression pattern of CAIX, which is particularly strong in the acid‐exposed surface cell region. As an alternative explanation for the observed phenotype, we hypothesized that the loss of CAIX may weaken the resistance of the gastric mucosa to acid and that the loss of parietal cells and the gland hyperproliferation may be secondary events in the wake of chronic acid damage. Therefore, the ability of *Car9* ^−/−^ surface cells to maintain the intracellular and the luminal pH immediately adjacent to the surface cells (‘juxtamucosal pH’) during a luminal acid load was examined *in vivo*. The changes in the cellular composition of gastric mucosa and its ability to secrete acid and mucus were investigated in Car9^−/−^ and WT littermates from newborn age to late adulthood.To further explore the causal relationship between acid damage, inflammation, parietal cell loss and foveolar hyperplasia, gastric proinflammatory cytokine levels and hedgehog expression as well as serum gastrin levels were measured. It has been recently established that a key element in conferring acid resistance to the gastric epithelium is the expression of the stomach‐specific claudin‐18 variants 18A2(1,2).^10^ Claudin 18A2 is expressed in the same location as CAIX, namely the basolateral membranes with predilection in the surface and chief cell region.^11^, ^12^ Therefore, we have also addressed the expression of claudin‐18A2 in the gastric mucosa of the CAIX‐KO mice and their WT littermates during the critical phase in which the barrier function is compromised. Finally, the ability of acid inhibitory strategies to prevent the observed morphological changes was assessed.

## Results

### Surface cell pH_i_ maintenance in *Car9* ^+/+^ and *Car9* ^−/−^ gastric mucosal surface cells *in vivo*

**Figure 1:**
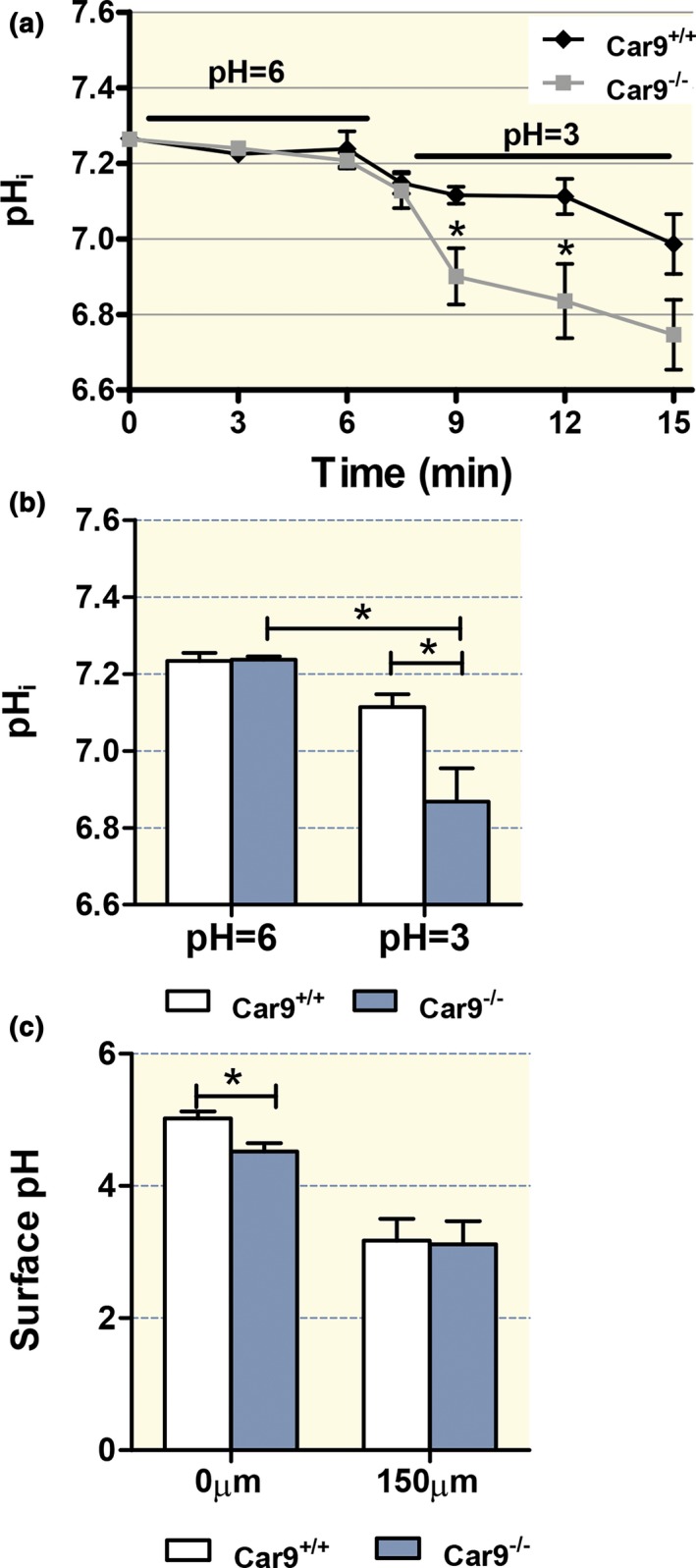
Intracellular and juxtamucosal pH maintenance is compromised in gastric surface cells in the absence of CAIX. (a) The pH_i_ of luminally acid‐exposed surface cells dropped significantly faster in anaesthetized *Car9* ^−/−^ mice compared to WT littermates (approx. 1.5–3 months of age). (b) No significant difference in steady‐state pH_i_ was assessed between gastric surface cells of *Car9* ^−/−^ and WT mice after equilibration with a luminal pH of 6 (left bars), but a significant pH_i_ difference was observed immediately after exposure to pH 3 (right bars). (c) The juxtamucosal pH was assessed at different distances from the surface cells with a pH of 3 in the luminal bulk solution. The extracellular pH was significantly higher close to the surface cells than at the edge of the mucus gel (compare left bars with right bars) in both genotypes, but it was significantly lower above surface cells of *Car9* ^−/−^ than WT mice. *n* = 4–7, *P < 0.05.

In vascularly perfused, exteriorized stomach of 1.5–3 mo/age anesthetized *Car9* ^+/+^ and *Car9* ^−/−^ mice, the surface cell pH_i_ was ˜7.25 during superfusion with luminal pH 6, with no difference between WT and KO (Fig. 1a,b left bars). Upon change to luminal pH 3 solution, the surface cell pH_i_ decreased significantly more rapid decrease of the surface cell pH_i_ was detected in the Car9‐/‐ cells (from 7.23 +/− 0.01 to 6.90 +/− 0.06 in 6 min) compared to the Car9+/+ cells (from 7.25 0.02 to 7.11 0.02 in 6 min). In the 3 months of age group, we also studied *Car9* ^+/+^ and *Car9* ^−/−^ mice that had been esomeprazole treated, and the respective values obtained were not significantly different from the respective *Car9* ^+/+^ and *Car9* ^−/−^ values without esomeprazole (data not shown). These results suggest that the decreased resistance to acid exposure is associated with the absence of CAIX expression and not by acid secretory inhibition.

### Juxtamucosal pH and mucus layer build‐up above *Car9* ^+/+^ and *Car9* ^−/−^ gastric mucosal surface cells *in vivo*

The juxtamucosal pH immediately adjacent to the epithelial cells was determined after superfusion with luminal pH 3 for 20 min in the exteriorized stomach of anaesthetized mice. Close to the epithelial cells, a significant difference in the juxtamucosal pH was observed between *Car9* ^−/−^ and *Car9* ^+/+^ stomachs (4.52 ± 0.12 vs. 5.02 ± 0.10), whereas such difference was not observed at distances of 150 *μ*m and more from the cells (Fig. 1c).

The dynamic build‐up of the mucus layer was determined fluorometrically with the use of fluorescent beads after removal of the adherent mucus by gentle suction (Fig. S1a), followed by superfusion with luminal pH 3 for 20 min. Despite the much longer mucus neck cell zone in the *Car9* ^−/−^ mucosa (Fig. S1c), the build‐up of the mucus layer was not significantly different between KO and WT mice (Fig. S1b). However, the firmly adherent mucus layer in Carnoy's fixed mucosa of mice without previous manipulation was thicker in *Car9* ^−/−^ mucosa (Fig. S1d). Thus, the ability of the surface cells to withstand a luminal acid load (Fig. 1) is decreased in KO stomach despite a thicker adherent mucus layer. The thicker adherent mucus may be a protective response or may be due to the impaired acid secretory rate and potentially diminished pepsin enzymatic activity, or the elongated mucus neck cell zone or all of the above.

### Changes in the morphology and the cellular composition of the gastric mucosa in *Car9* ^−/−^ mice from newborn to advanced age

**Figure 2:**
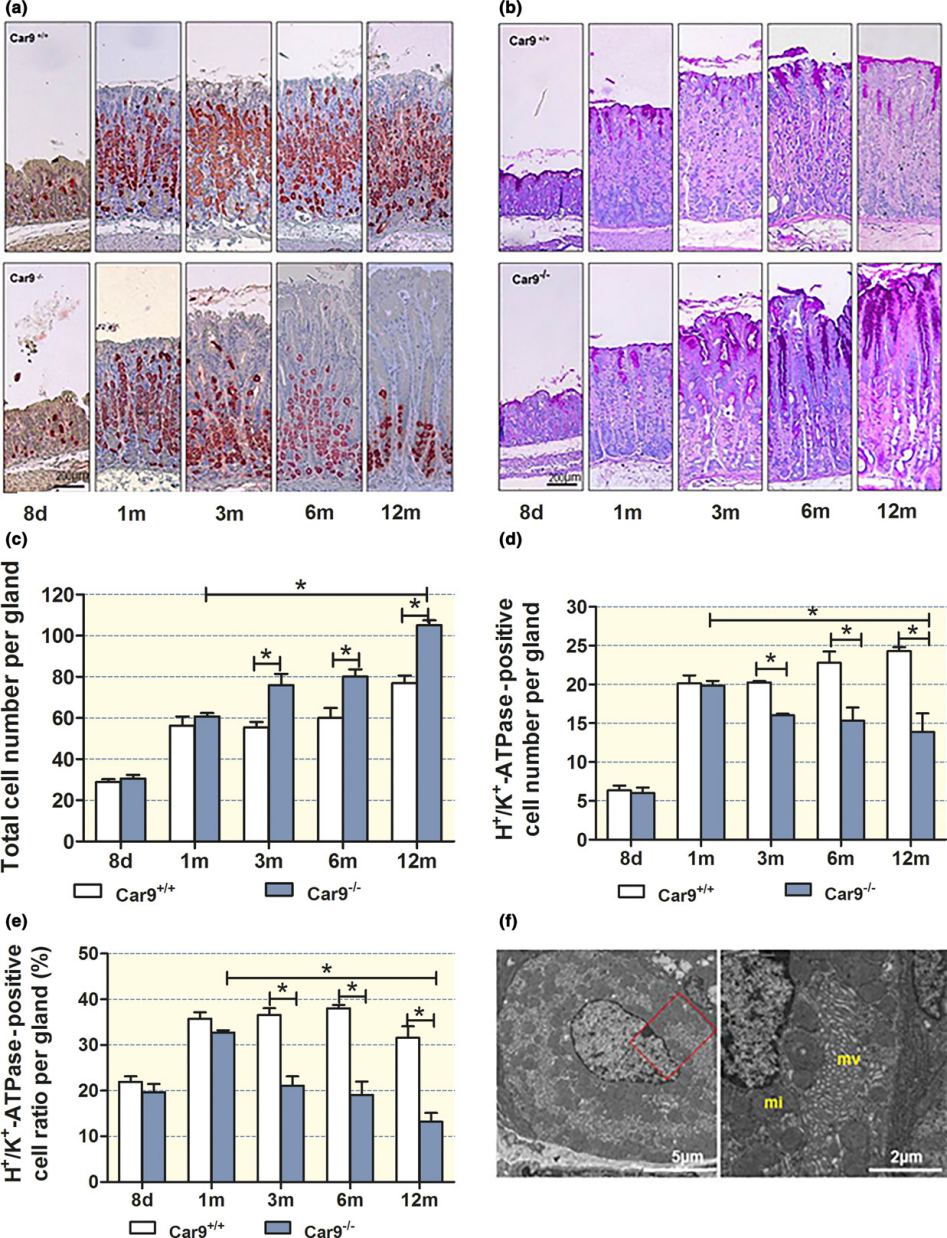
Morphology and cellular composition of the *Car9* ^−/−^ and WT gastric mucosa from newborn to advanced age. Distribution of (a) H^+^/K^+^‐ATPase‐positive parietal cells and (b) PAS‐positive mucous cells in the gastric glands over time. Upper panel: WT mucosa, lower panel: *Car9* ^−/−^ mucosa. (c) Total cell number per gland, (d) H^+^/K^+^‐ATPase‐positive cell number per gland and (e) the ratio of H^+^/K^+^‐ATPase‐positive/total cells per gland. A significant decrease in parietal cell number per gland/decrease in the parietal cell/total cell ratio was observed from 3 months of age. (f) Electron micrograph of a parietal cell at the base of a gland of a 9 month old *Car9* ^−/−^ mouse displays intact morphological feature. *mv* microvilli, *mi* mitochondria. *n* = 6, *P < 0.05.

No significant differences in the length of the gastric glands and the numbers of H^+^/K^+^‐ATPase‐positive cells per gland were observed between WT and KO gastric mucosae at 8 days and at 1 month after birth. At 1 month of age, a well‐developed gastric mucosa with surface, mucus neck, parietal and chief cells was present in WT and KO gastric mucosa (Fig. 2a,b and Fig. S2). This argues against a differentiation defect in the absence of CAIX expression. CAII mRNA expression levels were not different between WT and KO gastric mucosa both in non‐treated stomach and after esomeprazole feeding (Fig. S3).

In the mucosa of both WT and *Car9* ^−/−^ mice, the percentage of H^+^/K^+^‐ATPase‐positive cells increased from approx. 20 to 40% from day 8 to 1 month after birth. While it remained stable thereafter in WT mice (Fig. 2d, closed bars), a gradual decrease in parietal cell number to less than 20% of total cells within a gland was observed in KO mice (Fig. 2e). The gradual decline in numbers as well as a redistribution of the parietal cells to the lower part of the glands was accompanied by an expansion of the mucus containing neck and surface cell area, and a decline in the chief cells (Fig. 2a,b and Fig. S4). Chief cell number was not quantitated in our study, because it is well known that the differentiation of mucous neck into mature chief cells is dependent on the number and functional activity of the parietal cells and that chief cells transdifferentiate to SPEM (mucous cell metaplasia expressing TFF2/spasmolytic polypeptide) when parietal cells are lost.^13^

Even at 9 months, the remaining parietal cells in *Car9* ^−/−^ mice at the base of the glands were ultrastructurally indistinguishable from those in WT mice. They possessed abundant intact mitochondria and well‐developed secretory canaliculi (Fig. 2f), and showed no evidence of ultrastructural alterations that suggest either metabolic cell damage or morphological features similar to those described in acid transporter‐deficient parietal cells.^14^, ^15^, ^16^, ^17^

### Acid secretory capacity of isolated gastric mucosa in *Car9* ^+/+^ and *Car9* ^−/−^ mice

**Figure 3:**
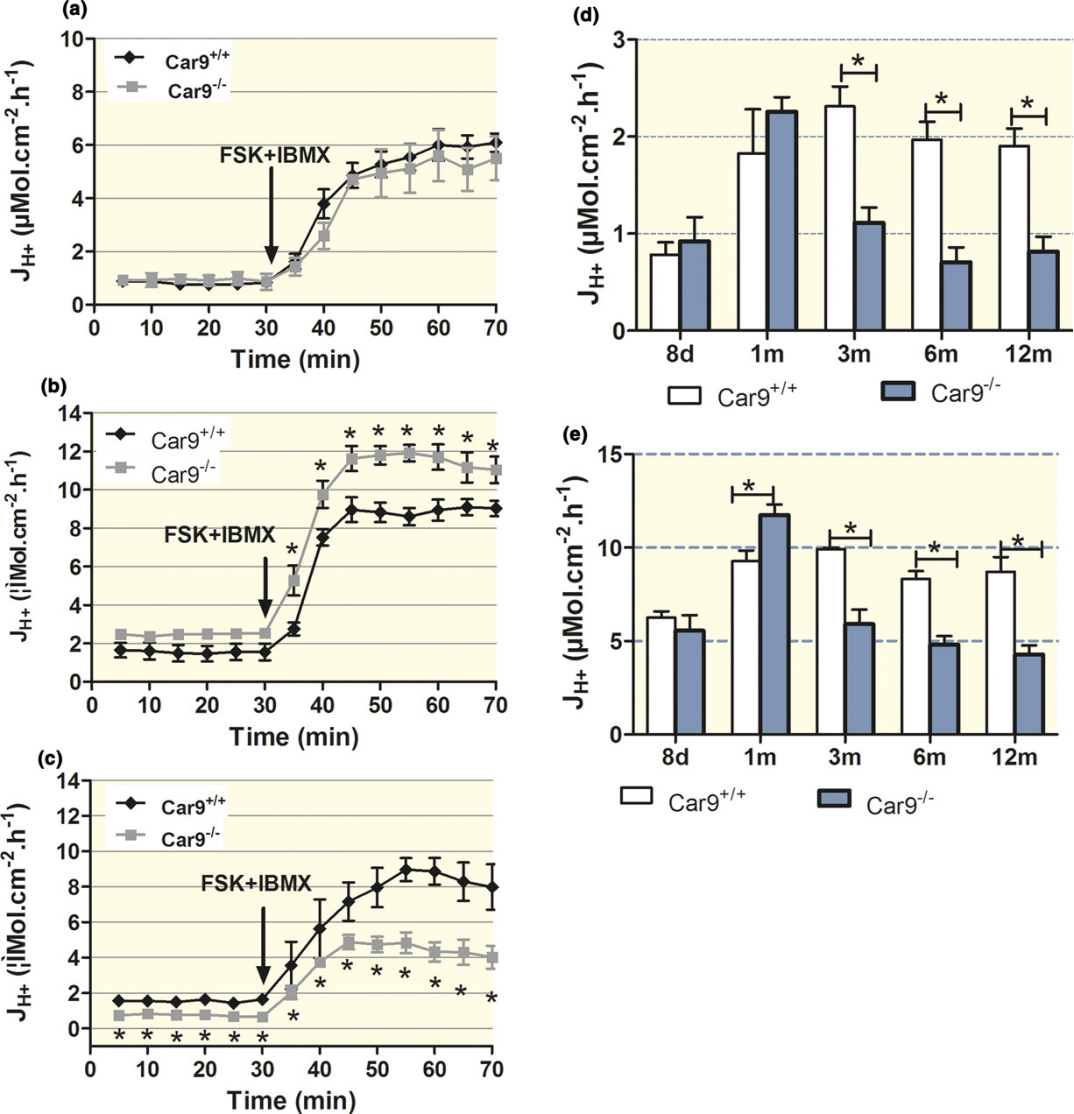
Acid secretory capacity declines in parallel with the decline in parietal cell number. Time course of acid secretory rates in isolated gastric mucosae of (a) 8‐day‐, (b) 1‐month‐ and (c) 6‐month‐old *Car9* ^−/−^ and WT mice. (d) Basal and (e) maximal acid secretory rates after stimulation with forskolin (FSK) and IBMX were significantly lower in *Car9* ^−/−^ mice than in WT up from 3 months of age. The decline in acid secretory capacity in the mucosa of *Car9* ^−/−^ mice is paralleled by the decline in parietal cell numbers (see Fig. 2). *n* = 6–7, *P < 0.05.

No difference was observed in basal secretory rates of the gastric mucosa in *Car9* ^+/+^ and *Car9* ^−/−^ mice at 8 days and 1 month after birth (Fig. 3a,b,d), and even a small but significant increase in the maximal acid secretory rate for the 1‐month‐old *Car9* ^−/−^ mice was detected (Fig. 3b,e). At higher age, both basal and maximal acid secretory capacity gradually declined in parallel with the decline of parietal cell numbers (Fig. 3c,e). Electrical parameters were measured in parallel and are shown in Table S1. Electrical resistance was not different between *Car9* ^+/+^ and *Car9* ^−/−^ mice at 8 days and 1 month after birth, but increased with age as parietal cell number declined and the thickness of the mucosa increased.

### Serum gastrin levels, parietal cell number and proliferative activity in *Car9* ^−/−^, *Car2* ^−/−^ and Kcnq1^−/−^ and the respective WT gastric mucosa

**Figure 4:**
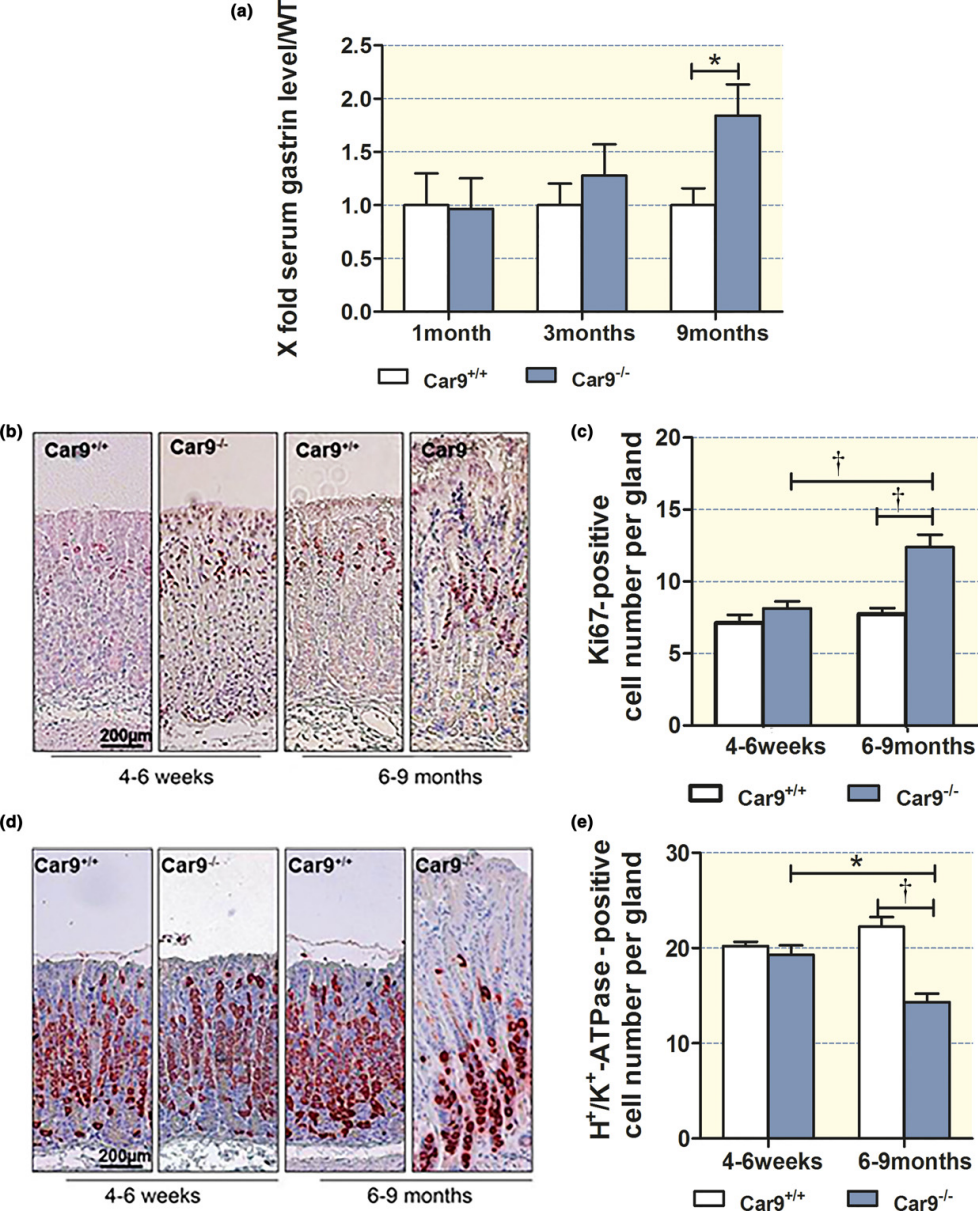
Inverse correlation between parietal cell number and serum gastrin levels and proliferative activity in CAIX‐KO mice. (a) Serum gastrin levels, (b, c) proliferating cells and (d, e) parietal cells in the gastric corpus mucosa of young (4–6 weeks) and adult (6–9 months) CAIX mice. *n* = 5, *P < 0.05*,* †*P < *0.01.

Fasting serum gastrin levels and acid secretory capacities were assessed in different mouse models with a partial or complete acid secretory defect at different ages (Fig. S4). Gastrin levels in *Car9* ^−/−^ mice were not different from those of WT mice at 1 month of age, but had almost doubled compared to WT at 9 months of age (Fig. 4a). Likewise, the proliferative zone of gastric glands in *Car9* ^−/−^ mice was not different from that of WT at 1 month of age, but significantly longer than WT with increasing gastrin levels (Fig. 4b,c). The parietal cell number displayed an inverse relationship with the gastrin level in *Car9* ^−/−^ mucosa (Fig. 4d,e).

In contrast, the elevated gastrin levels were associated with both elongated proliferative zones and an increase in parietal cell numbers in *Car2* ^−/−^ mice, which have a partial acid secretory defect from birth,^18^ or in Kcnq1^−/−^ mice, which have a complete acid secretory defect from birth,^15^ the elevated gastrin levels were associated with both elongated proliferative zones and an increase in parietal cell number (Fig. S4).

### Strong downregulation of the acid resistance factor claudin‐18 in *Car9* ^−/−^ gastric mucosae prior to the decrease in parietal cell numbers and development of foveolar hyperplasia

**Figure 5:**
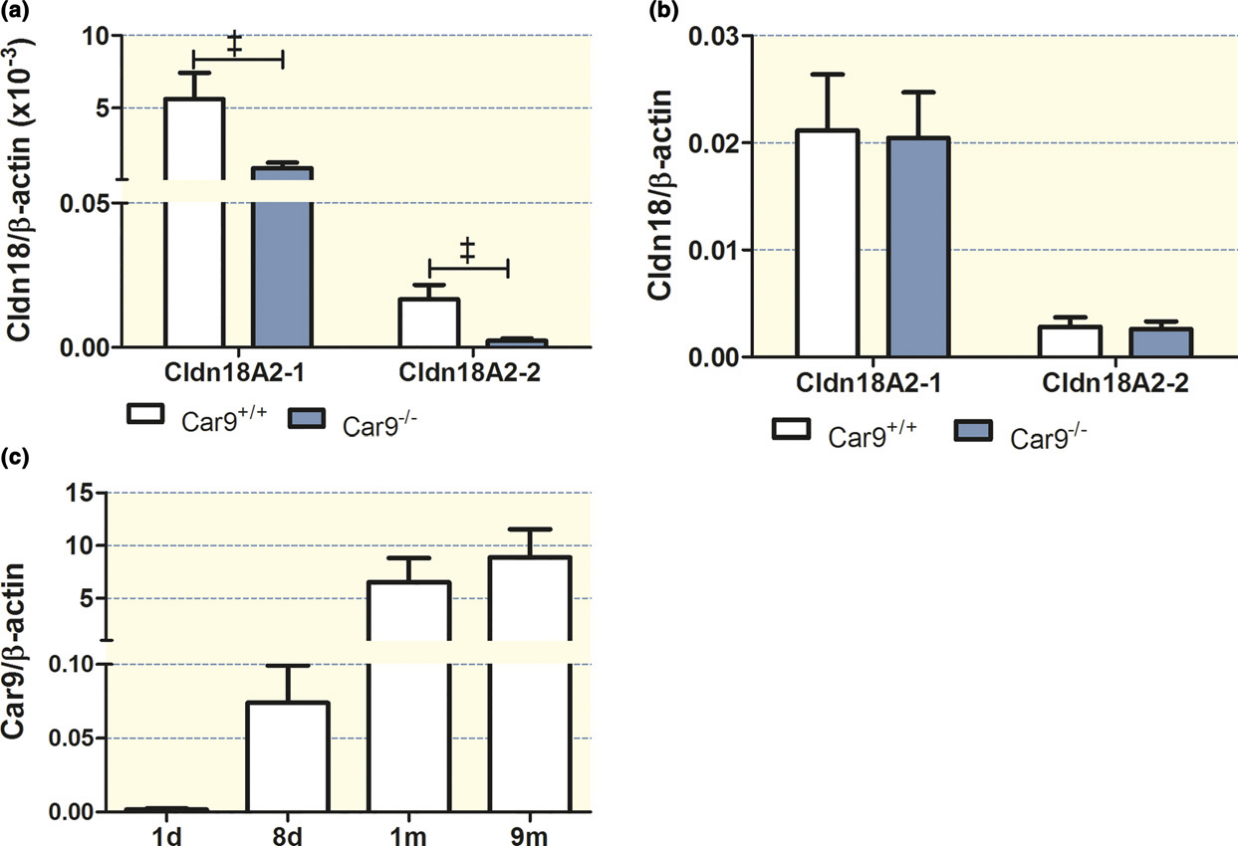
Claudin 18A2 and CAIX (Car9) expression in the gastric mucosa at 1 month of age. (a) mRNA expression of both claudin‐18A2 splice variants was significantly decreased in *Car9* ^−/−^ stomach compared to WT at the age of 1 month. (b) No change in the mRNA expression level of both claudin‐18A2 splice variants was observed between newborn *Car9* ^−/−^ and WT stomach. (c) mRNA expression of Car9 increases in WT stomach with increase of age (newborn to 9 months). *n* = 5 (*n* = 11 for newborn), &*P < *0.001.

The mRNA expression of both gastric claudin‐18A2 splice variants (18A2‐1 and 18A2‐2) was downregulated ∼ 4 fold in *Car9* ^−/−^ gastric mucosa at 1 month of age (Fig. 5a). In order to determine whether this decrease in claudin‐18 expression was due to an absence of CAIX during embryological development of the stomach, we compared the gastric mucosal claudin‐18 mRNA expression in newborn Car9^−/−^ and WT stomach. Claudin‐18A2‐1 and 18A2‐2 were highly expressed at birth (~8‐fold higher in relation to *β*‐actin than at 1 month of age), but not differently expressed in *Car9* ^−/−^ and WT littermates (Fig. 5b). We also determined the expression of CAIX in the WT mice at different age (Fig. 5c). CAIX expression was very low at birth and rapidly increased in parallel with the parietal cell number (Fig. 2d), and the acid secretory capacity of the gastric mucosa^14^, ^15^ as well as the development of intragastric acidity.^10^ The reciprocal post‐natal development of CAIX and claudin‐18A2 mRNA expression levels indicates that claudin‐18A2 mRNA expression is not dependent on CAIX mRNA expression. In contrast, it suggests that the lack of post‐natal CAIX upregulation in the gastric mucosa of *Car9* ^−/−^ mice precipitates a situation that results in a dramatic downregulation of claudin‐18A2 prior to the loss of parietal and chief cells.

### Cytokine and hedgehog mRNA expression levels

**Figure 6:**
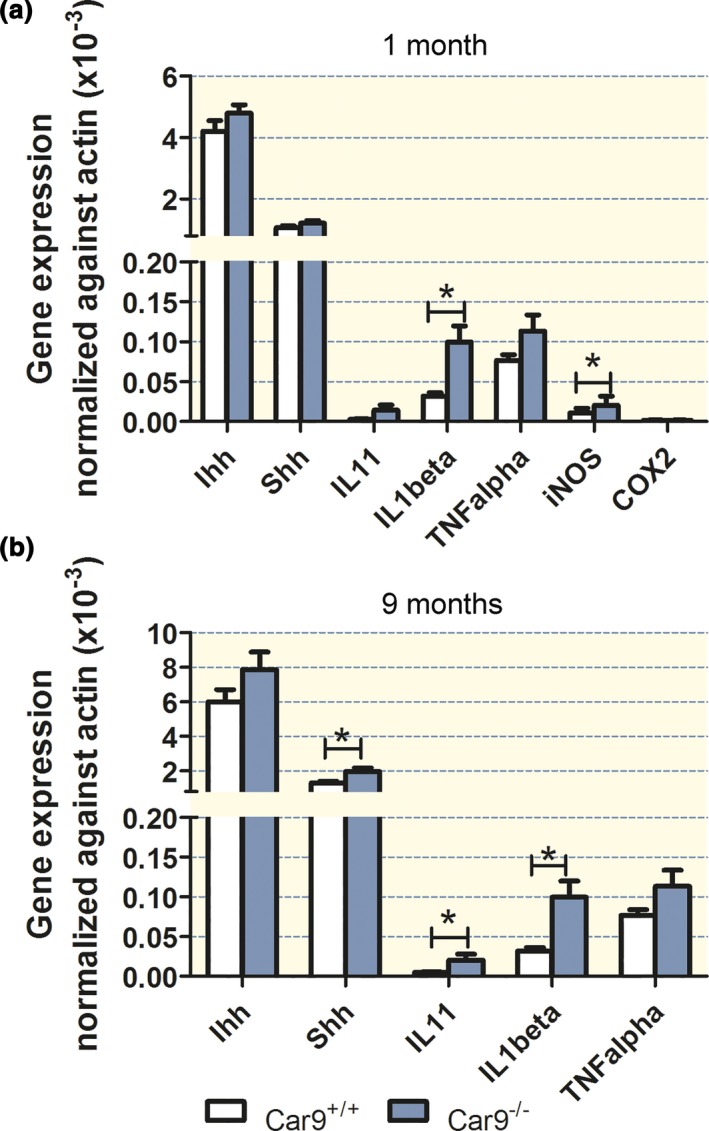
Cytokine and hedgehog expression levels in young and aged mice. (a) In 1‐month‐old *Car9* ^−/−^ and WT mice, a significant increase in IL‐1*β* and iNOS mRNA was observed, while the mRNAs of IL‐11, COX2 and the hedgehog proteins were not different from WT. (b) In aged (9 months old) mice, a significant increase in IL‐11, IL‐1*β* and Shh mRNA was observed compared to young mice. *n* = 6, *P < 0.05.

Sonic hedgehog (Shh) is an important regulator of gastric differentiation, while Indian hedgehog (Ihh) drives hyperplasia of surface mucous cells.^19^, ^20^ The proinflammatory cytokine IL‐1*β*, the inducible cyclooxygenase COX‐2 and the inducible nitric oxide synthase, on the other hand, are early markers of gastric injury.^10^, ^21^, ^22^, ^23^, ^24^ IL‐11 is a parietal cell cytokine that is released during gastric damage and induces atrophic gastritis.^25^ Inflammation is associated with decreased claudin‐18 expression in the airways^26^ and the stomach.^10^ To discriminate between abnormal development of parietal cells and acid damage‐induced parietal cell loss in the absence of CAIX, we measured the mRNA expression levels for Shh, Ihh, IL‐11, IL‐1*β*, COX‐2 and iNOS at 1 month (histologically normal mucosa) and at 9 months after birth [strongly altered mucosa with glandular elongation, hypergastrinaemia (see below) and parietal cell loss]. In *Car9* ^−/−^ mucosa, IL‐1*β* and iNOS were significantly increased compared to WT at 1 month of age (Fig. 6a), while IL‐11, COX‐2, Shh and Ihh expressions were not altered. This suggests that at 1 month, *Car9* ^−/−^ gastric mucosa has developed normally but is already under stress, evidenced by increased IL‐1*β* and iNOS expression. In contrast, newborn *Car9* ^−/−^ gastric mucosa did not show significantly increased markers for acute injury (Fig. S5).

In late adulthood, IL‐1*β* and IL‐11, as well as Shh expression, were significantly increased (iNOS and COX‐2 were not measured), likely reflecting advanced mucosal pathology and infiltration of the mucosa with non‐resident immune cells^27^ (Fig. 6b).

### Chronic submaximal acid inhibition attenuates the loss of parietal and chief cells in *Car9* ^−/−^ mice

**Figure 7:**
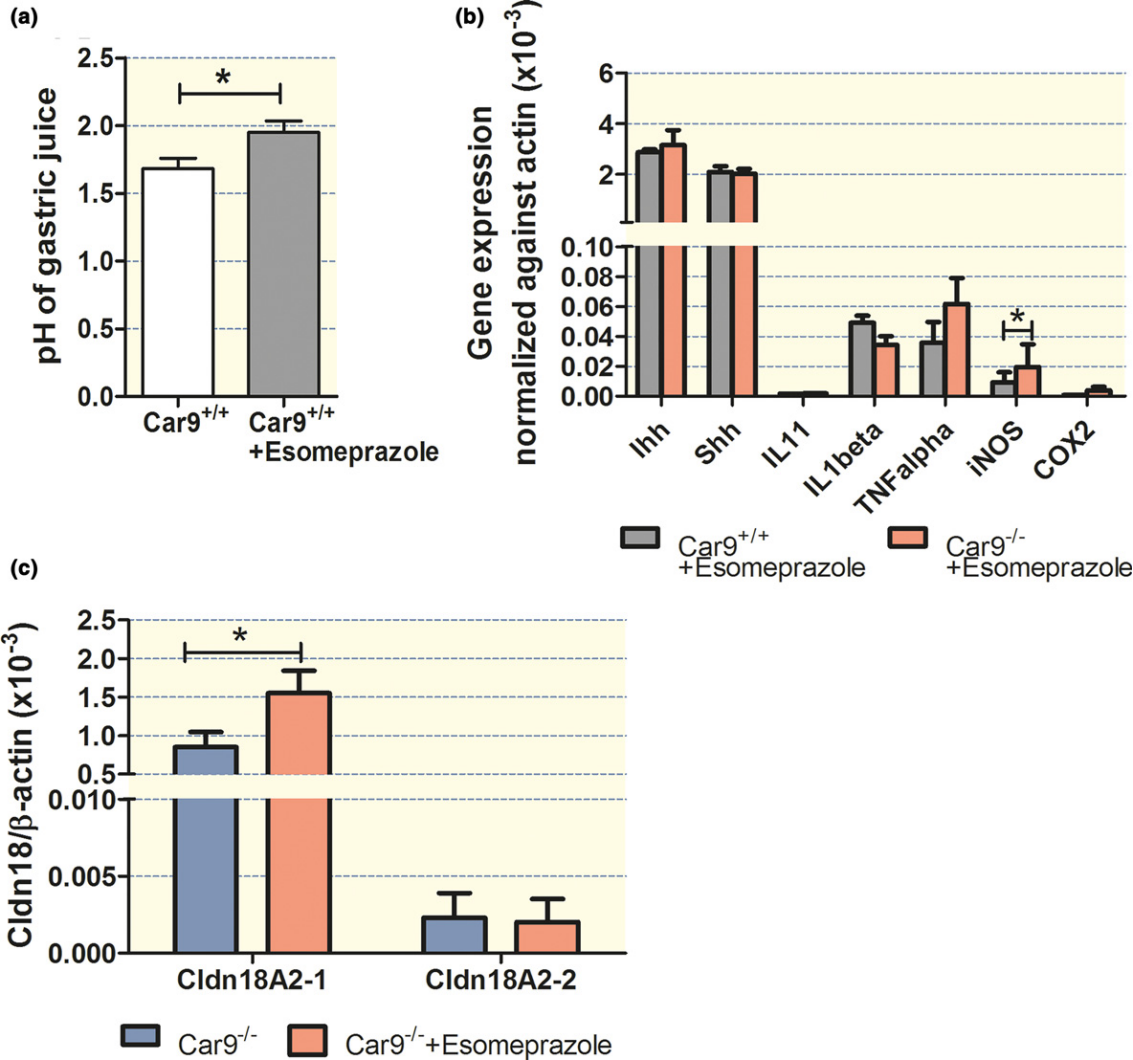
Chronic submaximal acid inhibition prevents IL‐1*β* increase but has only a minor effect and attenuates loss of parietal and chief cells in CAIX‐KO mice. Effect of an age‐adjusted step‐up scheme (see Materials and methods for dosing) of chronic esomeprazole feeding as indicated in the text on (a) pH of gastric juice, in histamine and pentagastrin‐stimulated, anaesthetized WT mice at 3 month/age to achieve a mild reduction in maximal acid output acidity. *n* = 5. (b) Cytokine and hedgehog expression levels at 1 month of age in *Car9* ^−/−^ and WT littermates at 1 month of age fed with 10 mg/kg chow during and post‐weaning. In contrast to non‐treated *Car9* ^−/−^ mice, the increase in IL‐1*β* mRNA was not observed, and the TNF‐*α* mRNA expression levels were significantly lower in WT and *Car9* ^−/−^ gastric mucosa, while iNOS mRNA expression remained same as in the non‐treated samples. (c) Esomeprazole feeding had a small but significant effect on claudin‐18A2‐1 mRNA expression (claudin‐18A2‐2 was not affected), but the expression levels were not restored to WT mucosa (see Fig. 5). *n* = 5, *P <* *0.05.

The above data suggest that critically important steps in inducing a stressed mucosal state in the *Car9* ^−/−^ stomach occur during the first month of life and may parallel the increase in gastric acidity during this time period. We therefore strove to develop a submaximal acid inhibition strategy from weaning to 3 months of age, to limit maximal gastric acidity, but to not result in bacterial overgrowth. Firstly, the amount of esomeprazole mixed with the chow was calculated based on the approximate daily intake of chow for mice, and the doses required to inhibit acid secretion in humans. The chronic esomeprazole feeding with the step‐up scheme as indicated in the method section was developed after studying different dosing regimens, started during weaning and applied for 1–3 months, and then, the maximal acid secretory rate in isolated gastric mucosa (age week 5 to week 12) as well as *in vivo* (for mice >8 weeks) was measured. As the results indicated that chronic feeding with 10 mg esomeprazole resulted in ~30% inhibition of the maximal acid secretory rate *in vitro* at 3 months of age (Fig. S6a), but did not significantly alter the intraluminal pH after *in vivo* stimulation with histamine and gastrin (data not shown), we developed a step‐up scheme for esomeprazole dosing (see Materials and methods section), which resulted in approx. 40% reduction in the maximal acid output in anaesthetized mice during stimulation with histamine and gastrin at 3 months of age (Fig. S6b). This resulted in a decrease in maximal acidity from ~pH 1.6 to 2.1 at 3 month of age (Fig. 7a).

Esomeprazole treatment (10 mg/kg chow) from weaning until 1 month of age (which has an unpredictable and possibly highly variable effect on acid suppression depending on how much solid food the pups take up during that time) resulted in lower mRNA expression levels of TNF‐*α* in both *Car9* ^+/+^ and *Car9* ^−/−^ gastric mucosa and prevented the increase in mucosal IL‐1*β* mRNA expression in *Car9* ^−/−^ gastric mucosa at 1 month of age, without changing the IL‐1*β* expression level in the *Car9* ^+/+^ (Fig. 7b, compare with Fig. 6a). The esomeprazole treatment did not alter the expression of iNOS. However, esomeprazole led to a significant increase in claudin‐18A2‐1 in the *Car9* ^−/−^ compared to non‐treated *Car9* ^−/−^, but not to the expression level of claudin‐18A2‐1 observed in WT stomach (Fig. 7c, compare with Fig. 5a). Esomeprazole did not affect claudin‐18A2‐1 expression in the WT stomach, nor claudin‐18A2‐2 in both *Car9* ^−/−^ and WT stomach.

**Figure 8:**
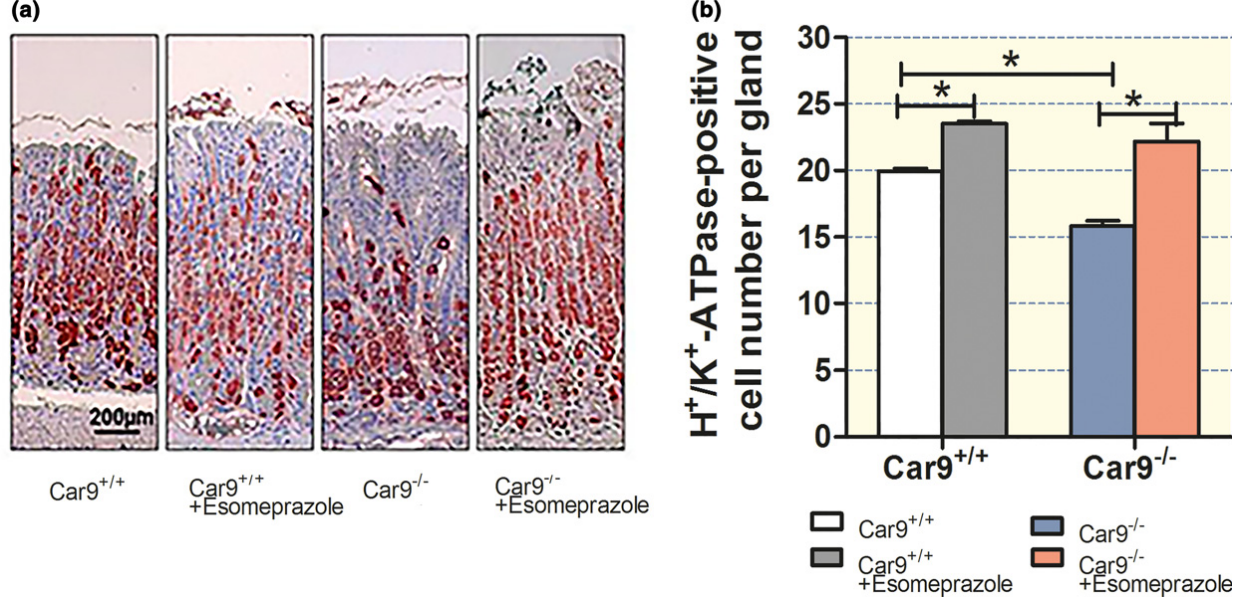
Chronic submaximal acid inhibition prevents loss of parietal and chief cells in CAIX‐KO mice. (a, b) Parietal cell number in the corpus of non‐treated and esomeprazole‐treated WT and *Car9* ^−/−^ mice. The significant decrease in parietal cell number between *Car9* ^−/−^ and WT mice was prevented by the reduction in maximal acid secretory rate. *n* = 4–6, *P < 0.05.

At 3 months of age, the esomeprazole step‐up scheme (10 mg/kg chow until day 30, 50 mg/kg to day 50 and 100 mg/kg to day 90)‐treated *Car9* ^−/−^ mice resulted in significantly reduced parietal cell loss than the nontreated *Car9* ^−/−^ mice (Fig 8a,b). A small increase in parietal cell numbers was also observed in WT mice (likely caused by mild hypergastrinaemia in both genotypes), but the significant difference in parietal cell number between *Car9* ^−/−^ and WT mucosa was suppressed. This strongly supports the concept that high intraluminal acidity or high acid secretory rates (with protons backfluxing from the neck of the glands via the weakened paracellular barrier) are the reason for the parietal cell loss and its sequelae in the *Car9* ^−/−^ mucosa.

## Discussion

CAIX is predominantly expressed in the basolateral membrane of the surface cells of the gastric mucosa,^8^, ^28^ while CAII, the other carbonic anhydrase isoform abundantly expressed in the gastric mucosa, is localized in the cytoplasm of surface as well as parietal cells.^29^ Parietal cells additionally express the mitochondrial CAV, which is also found in gastrin‐secreting cells.^30^ A role for CAIX in gastric acid secretion had previously been hypothesized based on an upregulation of CAIX in the *Car2*‐deficient stomach,^31^ and an interaction of CAIX with AE2.^32^ However, this concept had been abandoned, because the first report of the CAIX‐KO mouse phenotype described conspicuous morphologic alterations in the gastric mucosa, but no apparent change in gastric pH and serum gastrin levels.^8^ The authors therefore concluded that CAIX may be an essential component of gastric epithelial morphogenesis. This hypothesis cannot explain the gastric CAIX expression pattern, with the strongest expression in the surface cells,^8^ as well as the functionally and morphologically normal gastric mucosa of CAIX‐deficient mice at 8 days and 1 month of age observed in the present study. We therefore explored the hypothesis that CAIX functions as a pH control mechanism protecting the surface cells against luminal acid loads. The ability of the surface cells in CAIX‐deficient mice to withstand a luminal acid load, assessed by measuring the pH_i_ changes in SNARF‐5‐loaded gastric surface cells in vivo, was indeed decreased (Fig. 1). Even within the short timeframe that allows observation with the fluorometric dye technique, a significant difference in the acidification speed of CAIX‐KO vs WT surface cells was observed (Fig. 1a,b). This method requires prior loading of the surface cells with a pH‐sensitive dye and is able to focus exclusively on the intracellular milieu, but has limitations with respect to the observation time, because freshly secreted mucus scatters the fluorescence light. The values obtained for WT mice correspond with those previously published for rat stomach using this technique.^33^, ^34^

We had previously noticed that a high interstitial buffer capacity is essential for proper pH_i_ control of the gastric surface cells, because protons that are extruded through the basolateral membrane, via the Na^+^/H^+^ exchange, need to be rapidly removed from that location.^35^ The effect of interstitial buffer capacity on pH_i_ was detected even with a neutral pH in the lumen, but was strongly enhanced when the luminal pH was decreased. We had therefore hypothesized that cellular pH_i_ regulatory mechanisms are necessary for gastric acid resistance, but can only function optimally in the presence of high interstitial buffering capacity, augmented by bloodborne CO_2_/${\text{HCO}}_{3}^{-}$ . The protective effect of high plasma ${\text{HCO}}_{3}^{-}$ against acute gastric damage has been shown several decades ago.^36^ The gastric surface cells are not only the site of the strongest CAIX expression within the gastric mucosa, but also of the basolateral acid extruders NHE1 and NBC1.^2^, ^37^, ^38^ It has been shown that carbonic anhydrases form ‘bicarbonate metabolons’ with acid/base transporters, by binding close to the ion transporter's intra‐ or extracellular binding site for ${\text{HCO}}_{3}^{-}$ and augmenting the availability of this ion for transport.^9^, ^39^ The organization of the gastric microvasculature is such that it brings bicarbonate enriched blood extruded by parietal cells during acid secretion – the so‐called alkaline tide – directly to the surface cells.^40^ CAIX facilitates the rapid removal of protons to non‐toxic CO_2_, as long as the supply of ${\text{HCO}}_{3}^{-}$ is ensured. Thus, the CAIX in the gastric mucosa is ideally located to remove protons from the site of extrusion by the surface cells via their basolateral pH_i_ regulatory mechanisms.

Another parameter that has been shown to reflect intact gastric defence mechanisms is the assessment of the juxtamucosal pH directly above the epithelial cells.^41^, ^42^ In the present study, the juxtamucosal pH was significantly lower in the absence of CAIX in the presence of a luminal acid load, which may reflect lower rates of base permeation either via the apical membrane of the surface cells or via the anion‐selective tight junctional pathway (Fig. 1c).

The results are consistent with the concept of a role of CAIX as a regulator of the interstitial buffer capacity close to the gastric epithelial basolateral membranes. However, a surprising observation during the course of the study suggests that the lack of CAIX may not only interfere with epithelial pHi regulation, but actually result in a dramatic downregulation of the expression of claudin‐18A2. Claudin‐18 is by far the most abundant claudin expressed in the gastric mucosa and conveys the high resistance to protons.^10^, ^12^ Claudin‐18A2 was highly expressed in the newborn stomach, where CAIX was weakly expressed. CAIX expression increases dramatically in parallel with the increase in acid secretory capacity, whereas the relative claudin‐18A2 expression levels are higher in the newborn mucosa than at 1 month of age. Therefore, the lack of CAIX function in the post‐natal development of the gastric mucosa, most likely via insufficient acid handling, ROS production and proinflammatory cytokine release, downregulates claudin‐18 expression, thus further adding to the defect in acidity handling of the gastric barrier caused by the absence of CAIX expression.

The concept of a weakened gastric barrier to acid in the absence of CAIX expression also fits well with the morphological alterations seen in the stomach of *Car9* ^−/−^ mice. In the claudin‐18‐KO mouse, acid backflux from the gland lumen also causes parietal cell loss, but this occurs within the first days of life.^10^ A similar, albeit delayed, parietal cell loss followed by fundic hyperplasia and inflammatory cell infiltration occurs in the occludin‐KO mouse.^36^, ^43^ In the CAIX‐KO gastric mucosa, the only significant abnormality detected by us at one month of age was an increase in the gastric mucosal IL‐1*β* and iNOS expression, and the strong decrease in claudin‐18 expression (Figs 5 and 6). In claudin‐18‐KO newborns, an instillation of acid into the newborn stomach was associated with an increase in IL‐1*β* and COX‐2 expression in KO compared to WT mice,^10^ suggesting that IL‐1*β* and COX‐2 expression may rise secondary to acid backflux and prior to parietal cell loss. iNOS has been implicated in gastric damage as well.^23^, ^24^ The increase in IL‐1*β* and iNOS expression observed in the present manuscript may therefore be a sign of acid damage (Fig. 6). To test this hypothesis, we fed CAIX‐KO and WT mice with esomeprazole from the time of weaning to curb maximal acid secretory rates, which prevented the increase in IL‐1*β* and decreased the level of other proinflammatory cytokines. However, esomeprazole treatment attenuated but did not prevent the increase in iNOS and the downregulation of claudin‐18 in the KO mice (Fig. 7).

Progressive fundic hyperplasia was also observed in other mouse models of inflammation, such as the stomach‐specific IL‐1*β*‐overexpressing mouse,^27^ after chronic IL‐11 exposure,^25^ in *Helicobacter felis*‐infected mice,^44^ and in gastrin‐KO and omeprazole‐treated mice that develop gastric bacterial overgrowth.^45^ In these models, the inflammatory cytokines are believed to downregulate sonic hedgehog expression,^45^ an important parietal cell morphogen, leading to parietal cell demise and loss of gastric acidity.^19^, ^43^ The ensuing hypergastrinaemia then stimulates Indian hedgehog expression in the parietal cell‐depleted stomach and drives hypertrophy of the neck cell zone.^20^ In contrast, the stomach of young CAIX‐KO mice displayed normal hedgehog and IL‐11 expression (Fig. 6), and the increase in hedgehog and IL‐11 expression seen in CAIX‐KO gastric mucosae in late adulthood likely originates from infiltrating mesenchymal stem cells and maintains inflammation and fundic hyperplasia independently of the original injury.^46^ It is feasible, but has not been tested, that the loss of parietal cells in the above‐named mouse models is also due, in part, to a claudin‐18 downregulation. A strong decrease in the expression of claudin‐18, in particular in the surface cell region, was also reported in *Helicobacter*‐infected gastric mucosa.^10^ In contrast, the morphological alterations seen in mice with a strong [i.e. the Kcnq1 KO,^14^, ^47^ the Atp4*α* KO^48^] or a milder defect in the acid secretory apparatus [i.e. the CAII‐KO mouse^18^] differ from those found in the *Car9* ^−/−^ stomach. Although these gastric mucosae also develop glandular expansion and hypergastrinaemia, the production of parietal cells and the expression of parietal cell‐specific genes such as the H^+^/K^+^ ATPase are increased (Fig. S4), not decreased as seen in the CAIX‐KO stomach. Therefore, it is unlikely that CAIX deficiency affects gastric morphology via a defect in the acid secretory machinery.

What may explain normal acid secretion and parietal cell numbers in CAIX‐KO mice at 1 month of age, but an almost full expression of the phenotype by 3 months of age? We sacrificed CAIX mice during various times of the day from shortly after birth until 4 weeks of age and measured the acidity of both the stomach contents and the mucosal surface. The stomachs of the pre‐weaning mice were always full of coagulated milk and the pH was not below pH 4 in the first 2–3 weeks of life. In 4 week old mice, that had been separated from the mothers, the maximal acid secretory capacity was ~90% of that of 3‐month‐old mice and the intraluminal pH varied to as low as 2. An increase in proinflammatory markers and a decrease in claudin‐18 expression occur in the first months of life in the absence of CAIX. This indicates that even the immature gastric mucosa either secretes enough acid or is endangered by other acid moieties (byproducts of intragastric digestion of milk) to result in mucosal stress if CAIX is not expressed. However, the immature gastric mucosa of suckling pre‐weaning mice does not experience high luminal acid concentrations, and we speculated that acid damage by backflux from the glandular or gastric lumen starts to severely endanger the gastric epithelial cells after weaning. To test this hypothesis, the mice were subjected to chronic but partial inhibition of acid secretion until 3 months of age. Extensive experiments were performed to exactly define how to curb maximal acid secretion without causing hypoacidity to the extent that bacterial overgrowth occurs. This mild acid inhibitory strategy that we employed attenuated but did not prevent the increase in inflammatory markers and a decrease in claudin‐18 in the *Car9* ^−/−^ mucosa (Fig. 7), probably because such a strategy is ineffective before the young mice are weaned. However, it largely prevented the loss of parietal cells and resulted in a markedly lesser downward migration of the parietal cell zone in CAIX‐deficient mice (Fig. 8). This finding strongly undermines the concept that the parietal cell loss and regression to the lower gland area in the CAIX‐KO stomach is indeed caused by the intraluminal acidity in the neck of the glands and the gastric lumen.

Lastly, what may explain the apparent discrepancy between the results from Gut et al^8^ and our study? An ELISA for human gastrin determination had been used for the mouse sera by Gut *et al*., which also failed to show a difference between *Car9* ^*+/+*^ and *Car9* ^−/−^ mice in our study (the same strain as used by Gut *et al*.), but also failed to show differences between other mouse genotypes with known hypergastrinaemia, such as the *Kcnq1* ^−/−^ or the *Slc26a9* ^−/−^ mice. A more sensitive and specific radio‐immunoassay was therefore chosen for the measurement of murine serum gastrin. In addition, great care was taken to immediately place the drawn blood on ice, harvest the serum without interrupting the cold chain and immediately freeze in −80°C. Secondly, Gut *et al*. reported normal acid secretory rates based on the measurement of the intraluminal pH and the total acidity of the homogenized stomach.^8^ It is well known that the luminal pH does not correlate with the acid secretory rate and that in humans, low intragastric pH is measured after midnight, when acid secretory rates are low, but gastric emptying infrequent.^49^ In addition, the gastric pH values reported by Gut *et al*. are relatively high (compare to the values in Fig. 7a). In addition, total acidity of the homogenized stomach is, to our knowledge, not commonly used to define gastric acid secretory capacity. We also found that the assessment of maximal acid secretory rates in isolated chambered mucosa parallels the total number of parietal cells per gland better than any other measurement. We therefore carefully compared the maximal acid secretory rate in isolated *Car9* ^*+/+*^ and *Car9* ^−/−^ gastric mucosa with the parietal cell numbers and found a good correlation both in the WT and in the KO stomach (Figs 2 and 3). This underlines the fact that in the CAIX‐KO stomach, the remaining parietal cells have a normal capacity to store and engage tubulovesicles (Fig. 2f). Gut *et al*. reported no significant difference in parietal cell number, while we found a significant decrease (Fig. 2 and Fig. S2). A possible explanation for the non‐significant parietal cell loss might be that the area of the stomach examined was not from exactly the same region in the Gut *et al*.'s study or that the age of the examined mice varied considerably. Because the glandular length and parietal cell richness vary both from proximal to the distal end and between large and small curvature, we opened the whole stomach and took exactly the same area of the gastric mucosa from WT and KO for comparison. The gradual rise in serum gastrin levels is consistent with the progressive loss of parietal cells found in our study (Figs 2 and 4).

In summary, CAIX‐deficient gastric mucosal surface cells displayed a compromised ability to withstand a luminal acid load *in vivo,* an increase in proinflammatory cytokines, downregulation of claudin‐18 and parietal cell toxicity. Preventing strong gastric acidity from weaning until 3 months of age attenuated, but did not completely diminish the increase in inflammatory markers and decrease in claudin‐18 at 1 month of age, however it strongly reduced the loss of parietal cells in the subsequent 2 months. Important tasks for the future will be to delineate the exact sequence of events in the first month of life and the molecular mechanisms and consequences of claudin‐18 downregulation. An interesting question is also whether CAIX expression is stimulated by low pH, in addition to its regulation by hypoxia. The fact that CAII‐KO mice, which display significant respiratory acidosis,^50^ show an upregulation of CAIX expression along the whole gastric gland,^31^ may point in that direction.

## Materials and methods

### Animals breeding and chronic mild acid inhibition

*Car9* ^−/−^, *Car2*‐mutant and *Kcnq1* ^−/−^ mouse generation has been previously described.^8^, ^15^, ^31^ Mice were derived from the original strains described above and were congenic on the C57BL/6 background. The genotypes were verified by PCR. All studies were approved by institutional and independently by local governmental authorities. For the study of newborn gastric mucosal gene expression, the pregnant mothers were observed twice per day (approx. 8 a.m. and 5 p.m., and the newborn pups were killed immediately when detected (a few hours after birth). In selected experiments, graded doses of microencapsulated esomeprazole were mixed with the food from the time of weaning to reduce maximal acid secretory rates. 10 mg esomeprazole/kg chow was fed to day 30, 50 mg/kg to day 50, 100 mg/kg to day 90. The step‐up dosing schedule was chosen because of the lower acid secretory rates of young mice.^14^, ^15^ The efficacy of acid suppression was tested by maximally stimulating acid secretion both *in vivo* (Fig. 7 and Fig. S6b) and in isolated mucosa *in vitro* (Fig. S6a), and the goal was to curb maximal secretory rate and slightly elevate gastric pH, but not fully suppress acid secretion, to avoid hypergastrinaemia and bacterial overgrowth with maximal acid inhibition. To establish the esomeprazole feeding protocol, the mice were allowed to feed up to the day of the experiment for the *in vitro* experiments, and until midnight the day before the *in vivo* acid secretory experiments.

### Two‐photon confocal laser scanning microscopy *in vivo*

#### Measurement of pH_i_ in the surface epithelial cell of corpus mucosa

Mice were anaesthetized as previously described.^51^ After exteriorizing the vascularly perfused stomach, the mice were placed on the microscope stage of a two‐photon laser scanning microscope with an upright Leica TCS SP2 confocal microscope with a × 20 water immersion objective and a MaiTaiTi:sapphire‐pulsed laser (Spectra‐Physics, Darmstadt, Hessen, Germany). The exposed fundus/corpus region was loaded with SNARF‐5/AM and subsequently perfused with unbuffered saline, pH 6, followed by pH 3. The confocal scanning protocol has been described in detail previously.^51^

#### Measurement of juxtamucosal pH in the corpus mucosa

The mice were studied as described above, but the exposed corpus mucosa was incubated with unbuffered saline (pH 3.0) containing 10 *μ*mol LysoSensor™ Yellow/Blue DND‐160. LysoSensor was excited at 740 nm, and the emission was collected at 450 nm (410–475 nm) and 520 nm (485–610 nm). An *in vitro* calibration curve was made using solutions of different pH. Serial confocal xy fluorescence scans were performed every 30 *μ*m from the epithelium surface to the previously determined (see below) edge of the mucus layer and every 100 *μ*m to the solution surface, and was repeated at 10‐min intervals for 1 h.

#### Measurement of the thickness of the accumulated mucus in the corpus mucosa

The exposed mucosal surface was covered with 1 mL of unbuffered saline pH 3.0, and the mucus layer build‐up was assessed with the use of 15‐*μ*m fluorescent beads as described previously.^51^

### Light and transmission electron microscopy

Light microscopy and Ki67 immunohistochemistry were performed as previously described.^52^ Parietal cells were stained immunohistochemically with an anti‐H^+^/K^+^‐ATPase monoclonal antibody, according to the protocol of the manufacturer (Sigma Aldrich, Deisenhofen, Germany). The thickness of the firmly adherent mucus layer was determined after Carnoy's fixation of freshly excited gastric mucosa from non‐fasting mice,^53^ and then, the tissue was further processed for histological analysis as described.^52^ Comparison of the gastric morphology and cellular composition of *Car9* ^−/−^ and WT littermates was made at corresponding distances from the forestomach. Quantification of total cells and cell subtypes per gastric gland were determined as described before.^54^ For electron microscopy, samples of the corpus region were rapidly immersed in a fixative solution of 2.5% glutaraldehyde and 1.5% formaldehyde in HEPES buffer, pH 7.3, post‐fixed in OsO_4_ and prepared for electron microscopy using a standard protocol.^15^

### Ussing chamber experiments

Ussing chamber experiments were performed as described previously.^14^ Maximal acid secretory capacity was investigated by serosal application of 10 *μ* m forskolin (FSK) and 100 *μ* m IBMX, both purchased from Alexis Biochemicals (Lörrach, Germany).

### Serum gastrin levels

Mice were fasted overnight and killed by cervical dislocation, and the blood was taken immediately via heart puncture and placed on ice, centrifuged and the serum was collected. Serum gastrin levels were assayed by radio‐immunoassay in triplicate as previously described.^55^

### *In vivo* acid secretory studies

*In vivo* acid secretory rate was determined as described previously,^56^ with minor modifications: mice were fasted on grids that prevented coprophagy overnight, then anaesthetized by isoflurane inhalation as described in detail^51^; the abdomen was incised and the pylorus was ligated, pentagastrin was injected via the tail vein (16 *μ*g/kg), 1 mL of histamine in PBS was infused into the peritoneal cavity (3 mg/kg), and the abdominal incision closed. The mice were killed 2 h after pylorus ligation by cervical dislocation under anaesthesia. The gastric contents were collected, the pH was measured, and total acidity was determined by pH stat back titration.

### RT‐PCR for gastric proinflammatory cytokine and hedgehog expression

Gastric mucosa was scraped and RNA isolation was performed with RNeasy Mini Kit (Qiagen, Hilden, Germany); the integrity of the RNA was controlled with the QIAxcel system (Qiagen). Reverse transcription was performed with RevertAid Reverse Transcriptase (Thermo Fisher Scientific, Waltham, MA, USA). Real‐time PCR were carried out using Rotor‐Gene SYBR Green PCR Kit in the Rotor‐Gene Q Cycler (Qiagen). PCR extensions were performed at 60°C with 40 repeats. Data were analysed using Rotor‐Gene software and exported to Microsoft Excel. Relative quantification was carried out using actin and RPS9 as reference genes. The primer sequences, if not published elsewhere, are given in Table S2.

### Statistical evaluation

All results were expressed as mean ± SEM. ‘J_H+_’ represents the value of acid secretion. The data were analysed by anova if for multiple comparisons or Student's *t*‐test for paired samples. Results were considered significant at *P<0.05, †*P < *0.01, &*P < *0.001.

## Conflict of interest

The authors have no conflict of interest to disclose.

## acknowledgments

We thank Brigitte Rausch and Denise Renner for mouse breeding and genotyping. We thank Prof. Andrew Short, University of Edinburgh, for suggestions regarding clarity and style.

## Author contributions

T.L., X.L., B.R., A.K. S., K.A.M., A.S., Y. L., K. N., H. B.,. K‐H. H. and U. S. designed, performed and analysed experiments. G.G., H.B. and K‐H. H. provided tools, expert assistance and suggestions. T.L., B.R., K.N. and U.S. wrote the manuscript.

## Funding

The project was funded in part by DFG Grant SE460/19‐1 and by the Volkswagen Stiftung.

## Supporting information


**Table S1.** Potential difference (PD), short circuit current (*I*_sc_) and electrical resistance (*R*_t_) of isolated Car9^−/−^ and WT gastric mucosa.
**Table S2.** Primer sequences used for qtPCR.
**Figure S1.** Mucus layer build‐up and firmly adherent mucus layer in Car9^−/−^ and WT mice.
**Figure S2.** Giemsa staining of Car9^−/−^ and WT gastric mucosa at different age.
**Figure S3.** Car2 expression levels in young mice after chronic treatment with esometrazole.
**Figure S4.** Parietal cell number and proliferative zone in different knockout mouse models with compromised acid secretion and an increase in serum gastrin levels.
**Figure S5.** Cytokine expression levels in new‐born mice.
**Figure S6.** *In vitro* and *in vivo* acid secretory rates after chronic treatment with esometrazole.
