# Supplementary material for: Genetic ablation of carbonic anhydrase IX disrupts gastric barrier function via claudin‐18 downregulation and acid backflux
Source: Acta Physiol (Oxf). 2017 Oct 19;222(4):e12923. doi: 10.1111/apha.12923 (PMC5901031; doi:10.1111/apha.12923)

**Supplementary Table and Figures legends:**

**Supplementary Table 1: *Potential difference (PD), short circuit current (I_sc_) and electrical resistance (R_t_) of isolated Car9^‑/‑^ and WT gastric mucosa***

Potential difference (PD), short circuit current (I_sc_) and electrical resistance (R_t_) measured during the acid secretory measurements in isolated gastric mucosae of *Car9^‑/‑^* mice and WT littermates, at different ages. The mucosae of 8 day‑old mice were not stripped free of the muscle layer, which explains their higher resistance. n=4‑10, **P <* .05*, **P <* .01*.*

**Supplementary Table 2: *Primer sequences used for qtPCR***

**Supplementary Figure 1: *Mucus layer build-up and firmly adherent mucus layer in Car9^‑/-^ and WT mice***

Despite a longer mucus neck zone, the mucus layer build‑up, assessed by letting fluorescent beads settle on top of the freshly formed mucus gel at regular time intervals after suction (**a**), was not significantly different between *Car9*^‑/‑^ and WT mice (**b**). After Carnoy’s fixation (firmly adherent mucus), the surface mucus layer was thicker in the corpus mucosa of *Car9*^‑/‑^ than WT mice. (**c**) PAS staining of Carnoy’s fixed gastric mucosa; (**d**) thickness of adherent mucus layer. n = 4‑7, **P < .*05.

**Supplementary Figure 2: *Giemsa staining of Car9^‑/‑^ and WT gastric mucosa at different age***

The Giemsa stain is particularly suitable to distinguish parietal cells (light blue) from the chief cells (dark blue) in the base of the glands. The images show that both parietal and chief cell abundance is not different between Car9^‑/‑^ and WT gastric mucosa at 1 month of age, suggesting intact gastric cell lineage differentiation in young *Car9^-/-^* mice. Since loss of parietal cells results in chief cell loss^1^, the loss of both parietal and chief cells may both be a result of chronic acid damage.

**Supplementary Figure 3: *Car2 expression levels in young mice after chronic treatment with esometrazole***

No changes in the mRNA expression level of Car2 was measured in 1 month old *Car9^‑/‑^* and WT mice*.* Chronic treatment with esomeprazole did not alter the Car2 expression in both *Car9^‑/‑^* and WT mice. n=5.

**Supplementary Figure 4: *Parietal cell number and proliferative zone in different knockout mouse models with compromised acid secretion and an increase in serum gastrin levels*.**

(**a**) Serum gastrin levels were determined in other mouse models with partial (CAII), or complete (KCNQ1) loss of acid secretion. The bars show the relative increase in serum gastrin in full adulthood (6-9 months) n=3-5. (**b, c and d**) The proliferative activity, as judged by the number of Ki67‑positive cells, was increased in CAII and KCNQ1 KO gastric glands, correlating well with the gastrin levels. (**b,e and f**). The parietal cells also increased in both CAII KO and KCNQ1 KO gastric mucosa, confirming that elevated gastrin levels are the consequence of reduced acid secretory rates.

**Supplementary Figure 5: *Cytokine expression levels in new-born mice***

In new-born *Car9^‑/‑^* and WT mice, no changes in the expression level of IL-1β , iNOS and COX2 mRNA was observed n=11*.*

**Supplementary Figure 6: *In vitro and in vivo acid secretory rates after chronic treatment with esometrazole***

**(a)** Basal and FSK plus IBMX-stimulated acid secretory rates in isolated gastric mucosa from 3 months old WT mice fed with esomeprazole 10 mg/kg chow or 100 mg/kg chow. No esomeprazole was present in the bath solutions during the experiment. The slightly lower maximal stimulatory rates in the WT mice compared to those in Figure 3 are likely due to an experimentator less experienced in microdissection of gastric mucosa. It is obvious that in contrast to proton pump inhibitors present in the bath solutions, which completely abolish any acid secretion at 10-5M omeprazole, a residual secretory rate is present in mice treated with these feeding protocols. n=5 **(b)** Titratable acid output at 2 h post stimulation in anesthetized pylorus-ligated WT mice treated with the stepup esomeprazole feeding protocol as described in the methods section reveal that titratable acid output is only curbed, not abolished by the chronic esomeprazole feeding protocol (n=3).

| Age | Gene type |  | PD(mV) | |  | I_sc_  (µeq.cm^-2^.h^-1^) | |  | R_t_ (Ώ.cm^2^) | |
| --- | --- | --- | --- | --- | --- | --- | --- | --- | --- | --- |
|  |  |  | Basal | Peak  FSK+IBMX |  | Basal | Peak  FSK+IBMX |  | Basal | Peak  FSK+IBMX |
| **8-day-old** | Car9^+/+^(n=10) |  | 26.27±1.86 | 32.81±2.04 |  | 11.44±1.22 | 20.98±1.68 |  | 87.89±3.33 | 56.48±2.03 |
|  | Car9^-/-^(n=5) |  | 26.73±5.87 | 33.43±7.33 |  | 10.49±2.02 | 22.20±5.33 |  | 93.40±12.37 | 56.00±8.67 |
| **1 month** | Car9^+/+^(n=6) |  | 4.66±1,23 | 5.14±1.26 |  | 5.62±0.63 | 6.22±0.82 |  | 31.80±5.61 | 21.40±3.95 |
|  | Car9^-/-^(n=6) |  | 7.48±1,23 | 7.68±1.30 |  | 6.93±0.40 | 9.15±0.86 |  | 40.20±7.67 | 26.60±6.55 |
| **3 months** | Car9^+/+(^n=5) |  | 5.95±1.42 | 6.00±1.41 |  | 5.49±1.18 | 5.59±1.15 |  | 44.40±4.94 | 29.00±5.16 |
|  | Car9 ^-/-^(n=5) |  | 10.30±1.24 | 10.70±1.30 |  | 6.30±0.98 | 7.88±1.47 |  | 61.20±4.81**^*^** | 46.00±8.99 |
| **6 months** | Car9^+/+^(n=8) |  | 5.32±0.86 | 5.64±0.90 |  | 4.58±0.68 | 6.96±1.43 |  | 39.78±3.23 | 27.50±3.08 |
|  | Car9 ^-/-^(n=7) |  | 4.57±0.74 | 4.90±0.65 |  | 2.74±0.51 | 3.70±0.71 |  | 54.28±5.12**^*^** | 47.00±5.10* |
| **1 year** | Car9^+/+^(n=4) |  | 2.80±0.40 | 5.43±0.56 |  | 2.40±0.58 | 6.11±1.22 |  | 42.33±1.60 | 31.00±2.51 |
|  | Car9 ^-/-^(n=4) |  | 4.80±0.47* | 6.60±0.65 |  | 3.05±0.52 | 5.44±1.15 |  | 60.33±6.06* | 47.00±4.93* |

**Supplementary Table 1: *Potential difference (PD), short circuit current (I_sc_) and electrical resistance (R_t_) of isolated Car9^‑/‑^ and WT gastric mucosa***

**(a)** Basal and FSK plus IBMX-stimulated acid secretory rates in isolated gastric mucosa from 3 months old WT mice fed with esomeprazole 10mg/kg chow or 100mg/kg chow. No esomeprazole was present in the bath solutions during the experiment. The slightly lower maximal stimulatory rates in the WT mice compared to those in Figure 3 are likely due to an experimentator less experienced in microdissection of gastric mucosa. It is obvious that in contrast to proton pump inhibitors present in the bath solutions, which completely abolish any acid secretion at 10^-5^M omeprazole, a residual secretory rate is present in mice treated with these feeding protocols. n=5 **(b)** Titratable acid output at 2 h post stimulation in anesthetized pylorus-ligated WT mice treated with the stepup esomeprazole feeding protocol as described in the methods section reveal that titratable acid output is only curbed, not abolished by the chronic esomeprazole feeding protocol (n=3).

**Supplementary Table 2: Primer sequences used for quantitative PCR**

|  | **Sequence** |
| --- | --- |
| Ihh.for | 5´-AGAGCTCACCCCCAACTACA-3´ |
| Ihh.rev | 5´-CCAGTGAGTTCAGACGGTCC-3´ |
| Shh.for | 5´-AGCAGGTTTCGACTGGGTCT -3´ |
| Shh.rev | 5´-GCCACGGAGTTCTCTGCTT -3´ |
| IL11.for | 5´-CTGCAAGCCCGACTGGAA -3´ |
| IL11.rev | 5´-GATCACAGGTTGGTCTGGGG-3´ |
| IL1beta | QuantiTect Primer Assay, Qiagen |
| TNFalpha | QuantiTect Primer Assay Qiagen |
| iNOS.for | 5´-CGAAACGCTTCACTTCCAA-3´ |
| iNOS.rev | 5´-TGAGCCTATATTGCTGTGGCT-3´ |
| COX2.for | 5´-AACCGCATTGCCTCTGAAT-3´ |
| COX2.rev | 5´-CATGTTCCAGGAGGATGGAG-3´ |
| Cldn18A2-1. for | 5´-GTGAGTCCTCCCCTTCAAAGC-3´ |
| Cldn18A2-1. rev | 5´-GGCCAGCACTGGCTTTGGGTC-3´ |
| Cldn18A2-2. for | 5´-GAAGGGTTCCTCCTGCACACAGGT-3´ |
| Cldn18A2-2. rev | 5´-GGCCAGCACTGGCTTTGGGTC-3´ |
| Car2.for | 5´-CAAGCACAACGGACCAGA-3´ |
| Car2.rev | 5´-ATGAGCAGAGGCTGTAGG-3´ |

**Supplementary Figure 1**

**
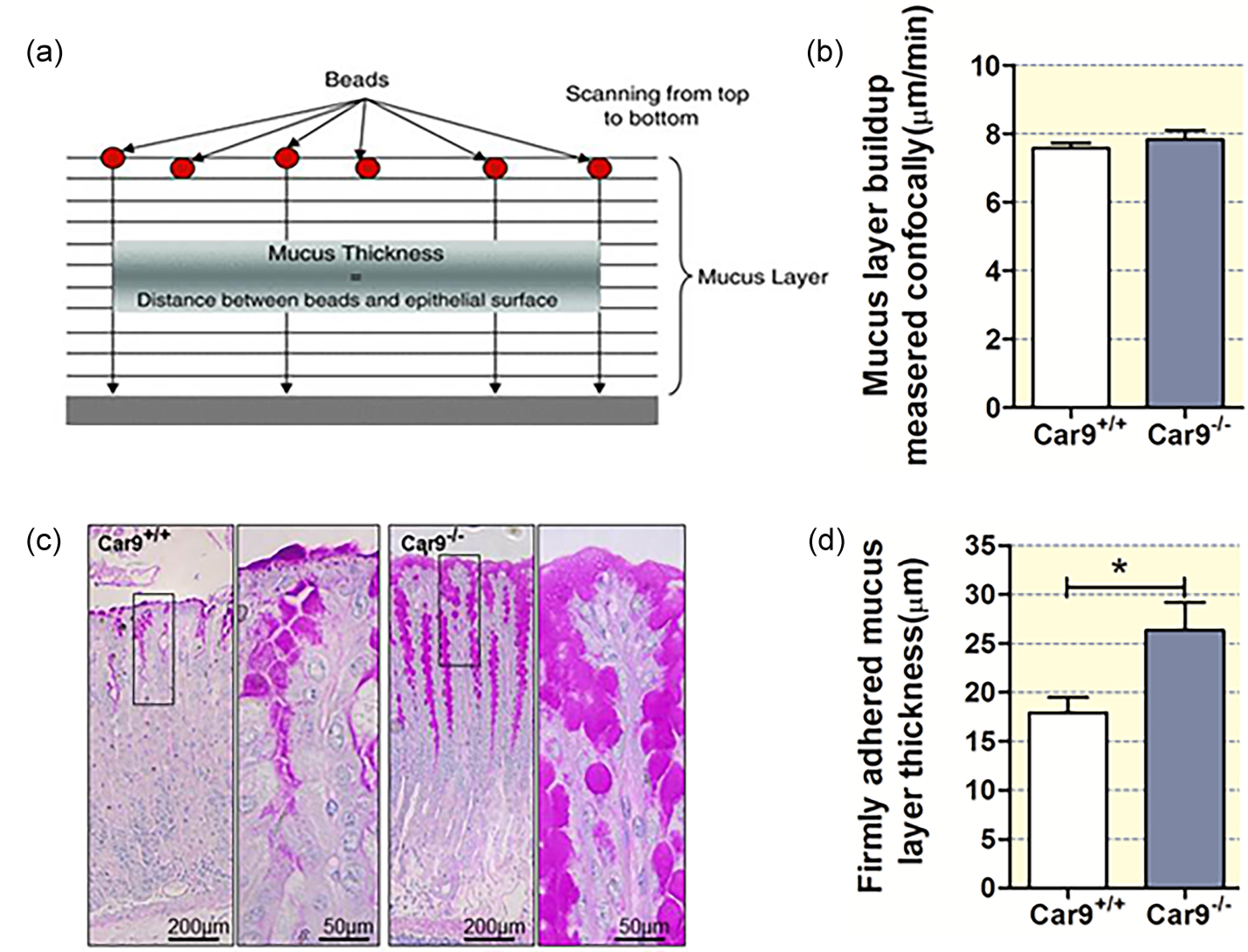
**

**Supplementary Figure 2**

**
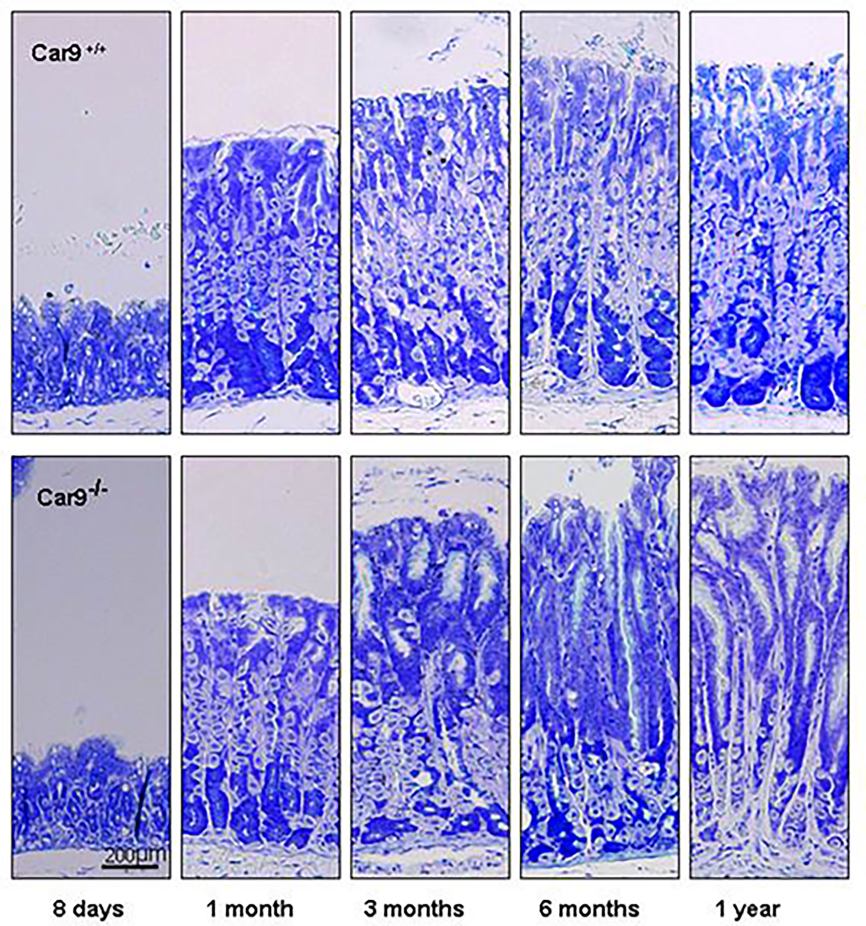
**

**Supplementary Figure 3**

**
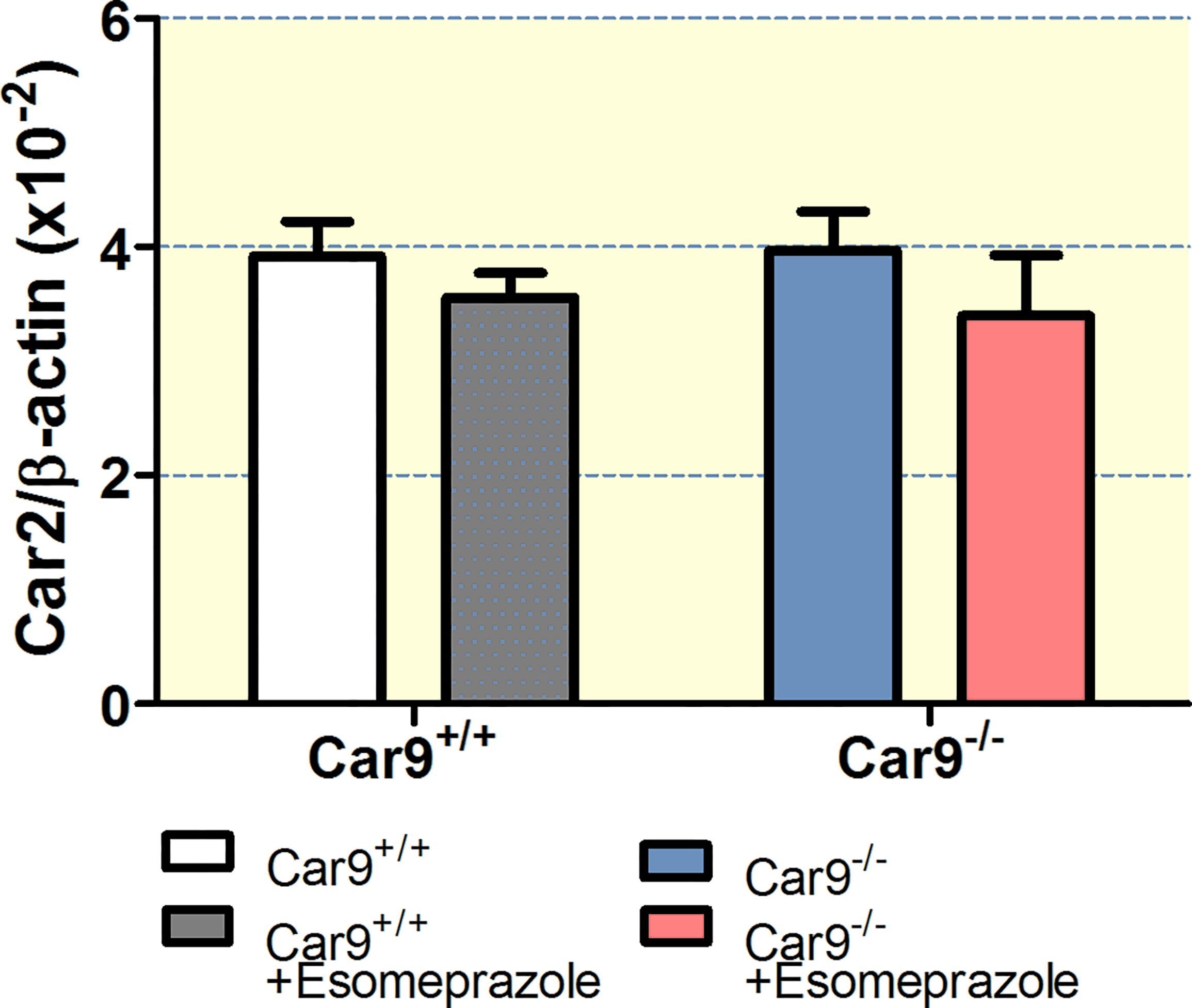
**

**Supplementary Figure 4**


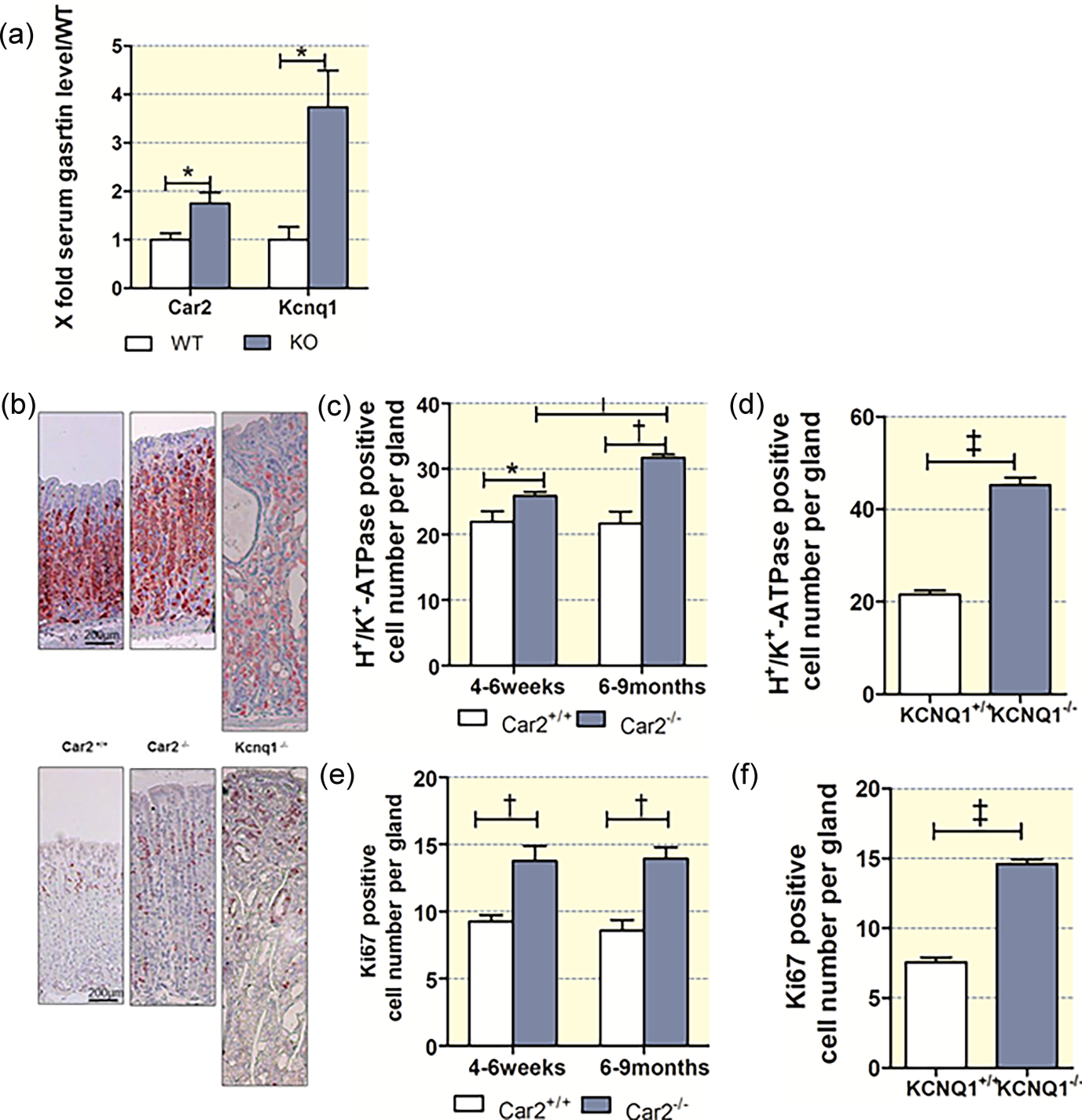


**Supplementary Figure 5**

**
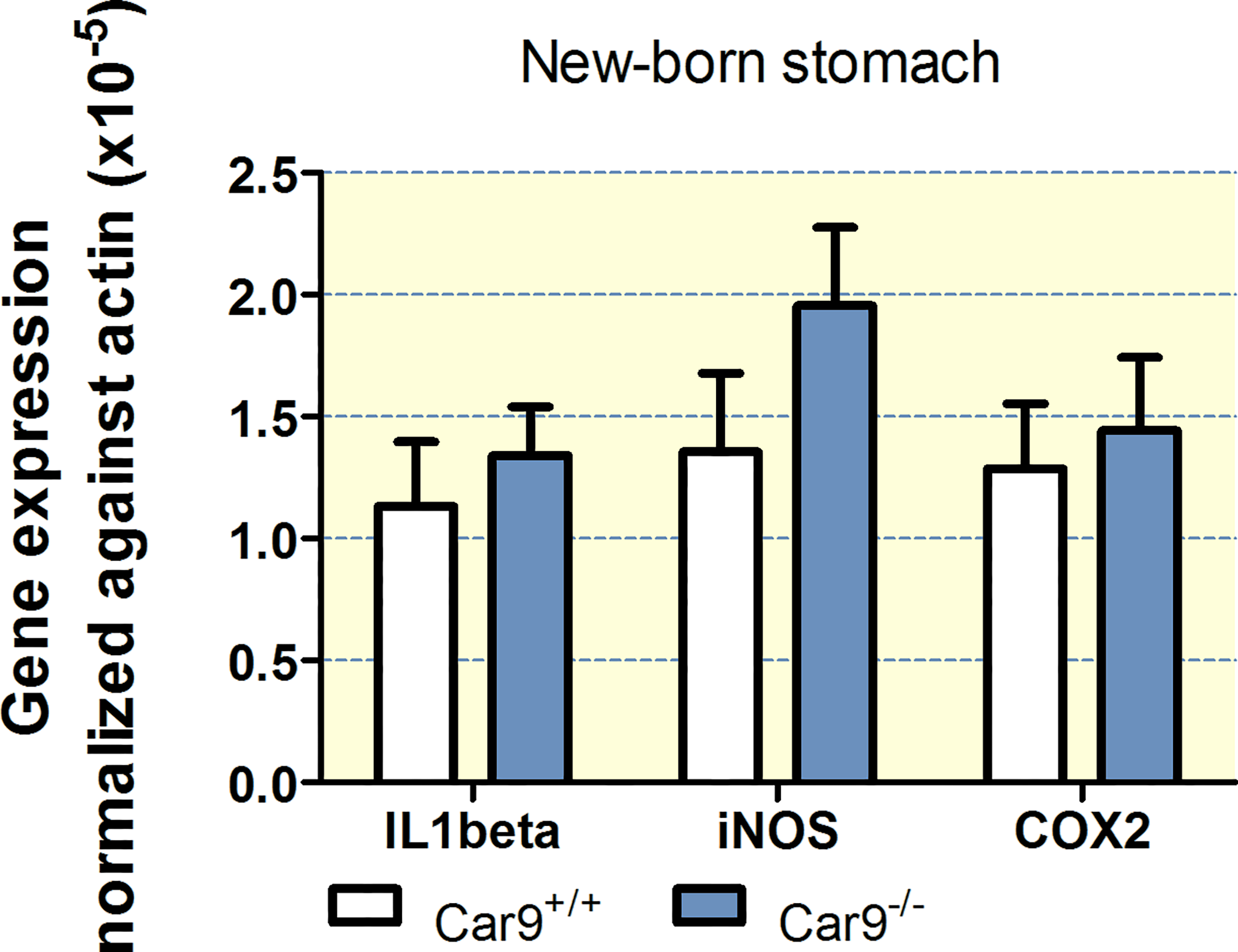
**

**Supplementary Figure 6**


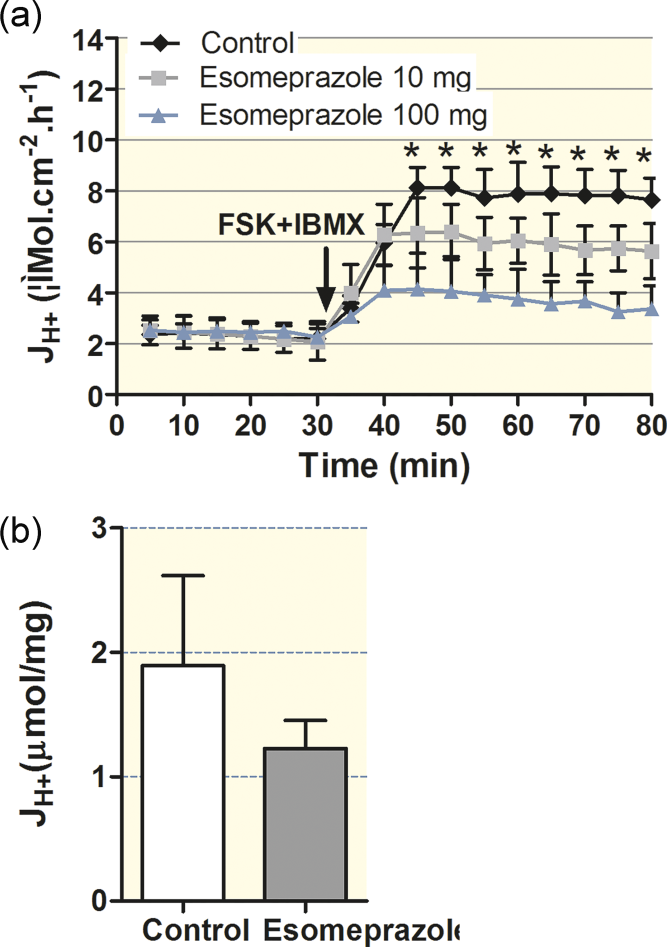

Supplement: Supplementary file 1 — Table S1. Potential difference (PD), short circuit current (I sc) and electrical resistance (R t) of isolated Car9−/− and WT gastric mucosa. Table S2. Primer sequences used for qtPCR. Figure S1. Mucus layer build‐up and firmly adherent mucus layer in Car9−/− and WT mice. Figure S2. Giemsa staining of Car9−/− and WT gastric mucosa at different age. Figure S3. Car2 expression levels in young mice after chronic treatment with esometrazole. Figure S4. Parietal cell number and proliferative zone in different knockout mouse models with compromised acid secretion and an increase in serum gastrin levels. Figure S5. Cytokine expression levels in new‐born mice. Figure S6. In vitro and in vivo acid secretory rates after chronic treatment with esometrazole. [file APHA-222-na-s001.docx]
